# Interplay between RNA‐protein interactions and RNA structures in gene regulation

**DOI:** 10.1002/2211-5463.70122

**Published:** 2025-09-12

**Authors:** Jenni Rapakko, Mauro Scaravilli, Minna‐Liisa Änkö

**Affiliations:** ^1^ Faculty of Medicine and Health Technology, Tampere University Finland; ^2^ Hudson Institute of Medical Research Melbourne Australia; ^3^ Department of Molecular and Translational Science Monash University Melbourne Australia

**Keywords:** CLIP, ribonucleoprotein (RNP), RNA structure, RNA‐binding protein, RNA‐protein interaction, SHAPE

## Abstract

Cellular RNAs are not linear single‐stranded stretches of nucleic acids as depicted in textbook cartoons but fold into secondary and tertiary structures through intra‐ and intermolecular base‐pairing. They also interact with proteins to form ribonucleoproteins (RNPs), the functional units of RNA in cells. Recent methodological developments utilising high‐throughput sequencing have enabled the detailed mapping of cellular RNA‐protein interactions and RNA structures. While methods for the direct determination of cellular RNP structures are still lacking, the integration of high‐throughput approaches and advancements with *in vitro* techniques such as cryogenic electron microscopy have provided insights into the functional significance of RNP structures. In this review, we will summarise the key methods used to probe cellular RNA‐protein interactions and RNA structures and then provide examples of how these approaches have led to an enhanced understanding of RNP structures in gene regulation and how this has also opened new avenues for drug development.

## Graphical abstract

Methodological advances in mapping transcriptome‐wide RNA‐protein interactions and RNA structures have started to uncover the potential of RNP conformations in gene regulation. Competing RNA–RNA, RNA‐protein and protein–protein interactions shape the compaction and function of RNPs throughout their lifetime and may provide novel therapeutic targets in various pathological conditions.

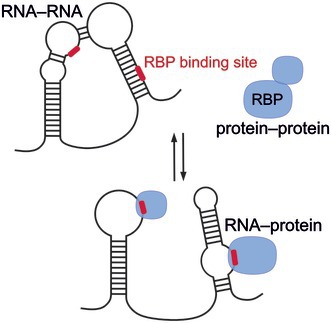

**Abbreviations**
2′‐OH: 2′‐hydroxyl group
4SU: 4‐thiouridine
5EU: 5‐ethyluridine
6SG: 6‐thioguanosine
AcIm: 1‐acetylimidazole
ADAR: adenosine deaminase acting on RNA
AI: artificial intelligence
AMT: 4′‐aminomethyltrioxsalen
APEX2: ascorbate peroxidase 2
APOBEC1: apolipoprotein B mRNA editing enzyme catalytic subunit 1
AR: androgen receptor
CLIP: crosslinking and immunoprecipitation
coCLIP: co‐localisation CLIP
CoSTseq: cotranscriptional structure tracking
CryoEM: cryogenic electron microscopy
DANCE‐MaP: deconvolution and annotation of ribonucleic conformational ensembles
DBCO: dibenzocyclooctane
DGCR8: DiGeorge syndrome critical region 8
DMS: dimethyl sulphate
dsRNA: double‐stranded RNA
eIF4A: eukaryotic initiation factor 4A
fCLIP: formaldehyde crosslinking and immunoprecipitation
FDA: US Food and Drug Administration
Fr‐iCLIP: fractionation individual‐nucleotide resolution crosslinking and immunoprecipitation
HCV: hepatitis C virus
HIF1A: hypoxia‐inducible factor 1A
hnRNP: heterogeneous ribonucleoprotein
HuR: human antigen R
HyPR‐MS: hybridisation purification of RNA‐protein complexes followed by mass spectrometry
icSHAPE: *in vivo* click selective 2′‐hydroxyl acylation analysed by primer extension
irCLIP‐RNP: infrared crosslinking and immunoprecipitation combined with mass spectrometry
KARR‐seq: kethoxal‐assisted RNA–RNA interaction sequencing
LNA: locked nucleic acid
miRNA: microRNA
ML: maximum likelihood
MS: mass spectrometry
NAI: 2‐methylnicotinic acid imidazolide
Nano‐DMS‐MaP: nanopore dimethyl sulphate mutational profiling
NMR: nuclear magnetic resonance
PADC: pancreatic ductal adenocarcinoma
PAR‐CLIP: photoactivatable‐ribonucleoside‐enhance crosslinking and immunoprecipitation
PARIS: psoralen analysis of RNA interactions and structures
P‐TEFb: positive transcription elongation factor b
RAP‐MS: RNA antisense purification coupled with mass spectrometry
RBM42: RNA‐binding motif protein 42
RBP: RNA‐binding protein
RIC: RNA interactome capture
RICK: capture of the newly transcribed RNA interactome using click chemistry
RIC‐seq: RNA *in situ* conformation sequencing
RNP: ribonucleoprotein
RP‐CONA: RNA pull‐down confocal nanoscanning
RRM: RNA recognition motif
RT: reverse transcriptase
SELEX: systematic evolution of ligands by exponential enrichment
SF3B: splicing factor 3B subunit 1
SHAPE: selective 2′‐hydroxyl acylation analysed by primer extension
SHAPE‐MaP: selective 2′‐hydroxyl acylation analysed by primer extension and mutational profiling
SHAPE‐seq: selective 2′‐hydroxyl acylation analysed by primer extension sequencing
snRNP: small nuclear ribonucleoprotein
SRSF3: serine/arginine‐rich splicing factor 3
ssRNA: single‐stranded RNA
STAMP: surveying targets by APOBEC‐mediated profiling
TRIBE: targets of RNA‐binding proteins identified by editing
UV: ultraviolet

Although it may seem apparent that RNA, having the intrinsic capacity to form base pairing interactions, can fold into diverse structures, the detailed characterisation of cellular RNA structures is only emerging with the exception of some highly structured noncoding RNAs such as transfer RNAs, ribosomal RNAs and microRNA (miRNA) hairpins. However, all RNA in cells is estimated to be compacted by at least ~50‐fold [1]. Recent work in yeast suggests that RNA folding starts as soon as the nascent RNA emerges at the site of transcription and is likely important for both the packaging and processing of RNAs [2]. At the same time, as the nascent RNA emerges at the site of transcription, it becomes bound by RNA‐binding proteins (RBPs) to form ribonucleoprotein (RNP) complexes that are the functional units of RNA in cells. While some structural RNAs have stable folds and the binding of RBPs such as the cap binding complex or poly(A) binding protein may be relatively stable, many RNA structures and RNA‐protein interactions are dynamic, with the potential to affect gene expression. Indeed, binding interactions between RNA and proteins mediate various RNA processing steps throughout the life of RNA from transcription to translation and finally RNA decay. These processes are essential for the regulation of various cellular processes including cell survival, differentiation and response to the environment (reviewed in [3]). Accordingly, alterations in RNA‐protein interactions are associated with several human disorders, such as neurodegeneration, autoimmune diseases and cancer [4, 5]. Similarly, RNA structures have important roles in gene regulation, affecting RNA biogenesis and function with links to human disease (reviewed in [6, 7]).

While there are powerful methods to study RNA‐protein interactions and cellular RNA secondary structures, less is known about their interplay. RNA folding and RBP binding are in constant competition in cells, and local RNA structures have the temporal advantage over long‐range interactions forming already during transcription. RNA structures may facilitate or inhibit protein binding to RNA, and RBPs may target a structure rather than a sequence. On the other hand, RBPs may act as RNA folding chaperones by bringing together distant complementary sequences or inhibit RNA base pairing by occupying complementary sequences. In this review, we will first introduce how cellular RNA‐protein interactions and RNA structures can be studied and then illustrate how they act together to mediate various steps in gene regulation with roles in disease pathogenesis and potential as targets for future therapies.

## How to study cellular RNA–protein interactions and RNA structures

*In vitro* methods dominated the study of RNPs and RNA structures for decades. The next‐generation sequencing era led to the development of innovative methods to probe transcriptome‐wide RNA‐protein interactions and RNA secondary structures in living cells. Methods assessing cellular RNA–RNA interactions have provided insights into the tertiary structures of RNAs, but the determination of higher order RNP structures in the cellular context remains a challenge.

### Mapping cellular RNA‐protein interactions

**Fig. 1:**
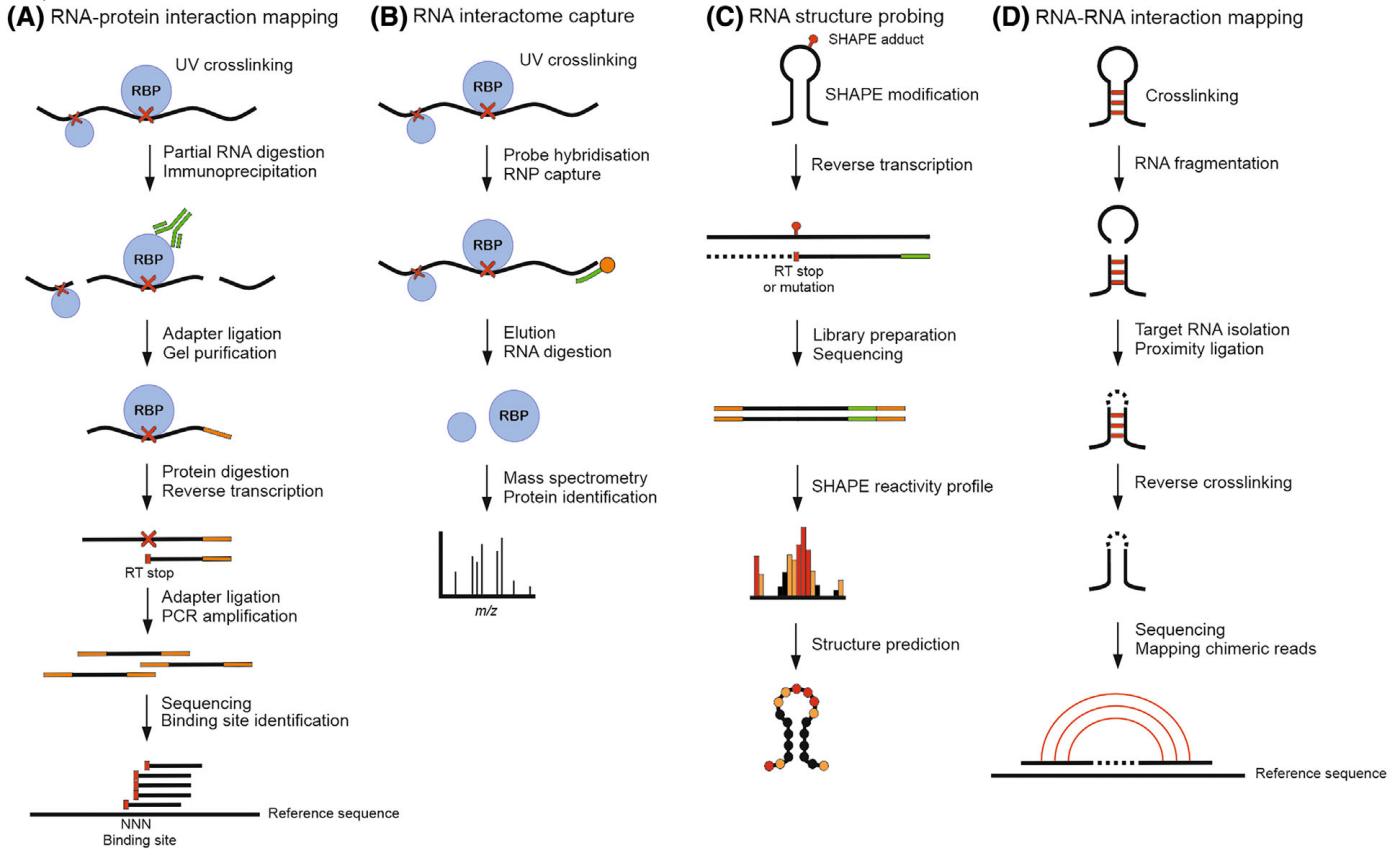
Schematic overview of methods to study RNA‐protein interactions, RNA structures and RNA‐RNA interactions. (A) RNA‐protein interaction mapping with crosslinking and immunoprecipitation (CLIP) to identify RNA‐binding protein (RBP) sites. UV crosslinked ribonucleoproteins (RNPs) are immunoprecipitated using antibodies targeting the protein of interest. Protein digestion leaves amino acid adducts at the crosslink sites, resulting in termination of reverse transcription (RT stops). RBP binding sites are identified based on the positions of RT stops in sequencing reads. (B) RNA interactome capture to identify RNA‐bound proteome. UV‐crosslinked RNPs are captured using probes specific to target RNA. Purified RBPs are identified with mass spectrometry. (C) RNA structure probing to determine RNA secondary structures. RNA is modified with Selective 2′‐hydroxyl acylation analysed by primer extension (SHAPE) reagent, which reacts with flexible nucleotides. RT stops or mutations, occurring adjacent to SHAPE‐modified nucleotides, are used to determine nucleotide reactivities, which are used as constraints in RNA secondary structure prediction. (D) RNA‐RNA interaction mapping to model intra‐ and intermolecular interactions. Covalent bonds between RNA base pairs are formed using a reversible crosslinker. Isolated double‐stranded RNA (dsRNA) fragments are ligated at one end, and crosslinking is reversed to obtain single‐stranded RNA (ssRNA). Each chimeric sequencing read represents a pair of interacting RNA regions.

The establishment of crosslinking and immunoprecipitation (CLIP) coupled to RNA sequencing transformed the study of cellular RNA‐protein interactions and is now widely used to map RBP target sites transcriptome‐wide *in vivo* (Fig. 1A). CLIP methods are based on the ability of short wave ultraviolet (UV) radiation to induce a covalent bond between two closely placed (~zero Ångström distance) aromatic rings that are found in the nitrogenous bases of nucleic acids and in several amino acids [8]. UV crosslinking is followed by RNase treatment and immunoprecipitation using antibodies targeting the protein of interest. The covalent bonds between RBP and the target RNA allow a stringent purification of specific RNPs [9]. The bound RNA fragments are characterised by next‐generation sequencing, and the high‐resolution mapping of the RBP binding sites is based on read termination induced by RBPs leaving amino acid adducts at the crosslink sites [10, 11, 12, 13]. In PAR‐CLIP (photoactivatable‐ribonucleoside‐enhanced crosslinking and immunoprecipitation), cells are treated with 4‐thiouridine (4SU) or 6‐thioguanosine (6SG) that are incorporated into the RNA prior to UV crosslinking [14]. The modified nucleosides have high photoreactivity, enhancing crosslinking efficiency. As the reverse transcriptase (RT) converts RNA containing the modified bases to cDNA, specific T>C and A>G mutations are introduced upon encountering 4SU and 6SG, respectively, corresponding to the crosslinked sites.

CLIP has also been adapted to characterise protein–RNA interactions in distinct subcellular compartments. In fractionation iCLIP (Fr‐iCLIP), chromatin, nucleoplasmic and cytoplasmic fractions are prepared from UV crosslinked cells and then subjected to iCLIP [15]. Alternatively, CLIP can be combined with proximity labelling used for high‐throughput protein localisation studies to assess RNA‐protein interactions in cellular compartments that cannot be readily separated by fractionation approaches. A genetically engineered ascorbate peroxidase (APEX2) that induces the oxidation of biotin phenol with hydrogen peroxide and generates short‐lived and highly reactive radicals can be directed to a specific cellular compartment by genetic fusion with a targeting peptide or protein [16]. In proximity‐CLIP, the RNA molecules are labelled with 4SU in cells expressing a localised APEX2 before the proximity labelling reaction takes place [17]. Lower energy UV light is used to crosslink proteins and RNAs similar to PAR‐CLIP, resulting in the formation of biotinylated and crosslinked protein–RNA complexes. The RNPs are selectively isolated with streptavidin affinity purification to characterise the subcellular RBPome by high‐throughput proteomics and transcriptomics analyses. In colocalisation CLIP (coCLIP), a localised APEX2 is used in the absence of 4SU labelling, generating biotinylated proteins in the compartment of interest [18]. CLIP targeting an RBP of interest is followed by a coCLIP step using streptavidin affinity purification. By comparing the whole cell CLIP to the coCLIP, a map of specific subcellular RNA‐binding interactions can be deduced for the protein of interest.

As an alternative to crosslinking‐based methods, targets of RNA‐binding proteins identified by editing (TRIBE)/HyperTRIBE and surveying targets by APOBEC‐mediated profiling (STAMP) involve fusing an RBP to the catalytic domain of a deaminase enzyme (adenosine deaminase acting on RNA, ADAR, in the case of TRIBE, and apolipoprotein B mRNA editing enzyme catalytic subunit 1, APOBEC1, in the case of STAMP) [19, 20, 21]. Expression of the fusion proteins in cells results in A‐to‐I editing in TRIBE and/or C‐to‐U editing in STAMP at residues close to RNA‐binding sites. By co‐expressing two RBPs fused to different RNA editing enzymes, the binding sites of two distinct RBPs can be mapped simultaneously (TRIBE‐STAMP) [22].

RNA‐centric methods to study cellular RNPs have greatly expanded the RNA‐bound proteome by identifying new RBPs (Fig. 1B) [3, 23, 24, 25]. Similar to the protein‐centric CLIP methods, UV crosslinking is used to stabilise RNPs prior to purification by target RNA‐specific probes. In RNA interactome capture (RIC), poly(A) tailed RNAs are captured using oligo(dT) beads and the purified RBPs are identified with mass spectrometry (MS) [23]. Capture of the newly transcribed RNA interactome using the click chemistry (RICK) method expanded the analysis of RNA interactomes to nonpolyadenylated RNAs by first labelling RNAs with 5‐ethyluridines (5EU) that can be biotinylated and recovered using click chemistry [24]. Specific RNP capture with antisense locked nucleic acid (LNA)/DNA mixmers makes use of a single 20‐mer probe to capture specific RNA species [25]. LNA modifications increase the probe affinity to the complementary target as well as the melting temperature, supporting selective hybridisation conditions. To capture specific RNPs, RNA molecules of interest can also be genetically engineered to contain a tag, such as a streptavidin‐binding RNA aptamer for RNP isolation by affinity purification [26]. However, the tag can disturb RNA secondary structures and interactions with RBPs [27]. RNA antisense purification coupled with MS (RAP‐MS) uses biotinylated antisense probes of ~120 nt in length that are captured with streptavidin beads [28]. Hybridisation purification of RNA‐protein complexes followed by MS (HyPR‐MS) was designed to target multiple RNAs simultaneously in a single experiment with biotinylated antisense probes [29]. In nonisotopic ligation‐based ultraviolet‐light‐induced crosslinking and immunoprecipitation combined with MS (irCLIP‐RNP for short), CLIP and interactome capture methods were combined to characterise the nature and dynamics of multiprotein assemblies on RNA [30].

The main challenges of the antisense probe‐based methods are related to the low copy number of the target RNA. Many of the adaptations of CLIP require a large number of cells as the starting material, limiting the analysis to cultured cells and large tissue samples. The UV light can only penetrate a few cell layers, which further limits samples that can be assessed by these methods. Because UV crosslinking is inefficient and UV light does not efficiently crosslink proteins to double‐stranded RNA [31, 32], chemical crosslinkers have been used in some RNA‐protein interaction applications such as formaldehyde CLIP (fCLIP) [33] but most chemical crosslinkers are not specific to RNA.

### Chemical probing for determination of cellular RNA secondary structures

Because single‐stranded RNA regions react more readily than regions engaged in base pairing interactions, differences in RNA reactivity can be exploited in chemical probing methods as a proxy for RNA secondary structure. The selective 2′‐hydroxyl acylation analysed by primer extension (SHAPE) quantitatively maps local RNA structures at a single nucleotide resolution (Fig. 1C) [34]. The technique takes advantage of the reactive sensitivity of the ribose 2′‐hydroxyl group (2′‐OH) to local nucleotide flexibility, which enables the acylation of 2’‐OH by SHAPE reagents, resulting in the formation of 2′‐O‐adducts (Fig. 1C). Proximity of the neighbouring negatively charged 3′‐phosphodiester group decreases the reactivity of the ribose 2′‐OH group. Thus, 2′‐OH reactivity reports whether a given nucleotide is accessible or constrained by base pairing (or to some extent tertiary interactions). Compounds commonly used in RNA structure probing are summarised in Table 1.

**Table 1:** Compounds for chemical probing of RNA secondary structure.

Compound	Full name	Reactivity	Properties	References
NMIA	*N‐*methylisatoic anhydride	2′‐OH (all bases)	*In vitro*, slow‐acting and short‐lasting reactivity	[34]
NAI	2‐methylnicotinic acid imidazolide	2′‐OH (all bases)	*In vitro* and *in vivo*, long‐lasting reactivity and high cell‐permeability, quenching step required	[35]
NAI‐N3	2‐methylnicotinic acid imidazolide azide	2′‐OH (all bases)	*In vivo*, biotin coupled azide group allows selective purification of modified RNA	[36]
1 M7	1‐methyl‐7‐nitroisatoic anhydride	2′‐OH (all bases)	*In vitro* and *in vivo*, fast‐acting and self‐quenching	[37]
AcIm	1‐acetylimidazole	2′‐OH (all bases)	Produces compact 2′‐*O*‐ adducts to allow identification of modified RNA by Nanopore sequencing.	[38]
DMS	Dimethyl sulphate	N3 position of C and N1 of A	*In vivo* and *in vitro*, robust chemical reactivity, fast cell‐permeability	[39]

Initially, primer extension followed by electrophoresis was used to map RT termination sites at the 2′‐O‐adducts [40]. However, this low‐throughput analysis was limited to individual, relatively short RNAs. *In vivo* click SHAPE (icSHAPE) was developed for high‐throughput interrogation of transcriptome‐wide RNA structures in living cells [36]. A 2‐methylnicotinic acid imidazolide (NAI) derivative, NAI‐N_3_, containing an azide group to which a biotin can be coupled, enabled selective purification of modified RNA molecules with streptavidin beads. Because RNAs with SHAPE adducts are separated from the total RNA pool, icSHAPE does not require extensive read coverage, unlike many other high‐throughput SHAPE methods. SHAPE sequencing (SHAPE‐Seq) was first introduced for *in vitro* transcribed RNAs [41] but was later applied to RNA pools extracted from cells (SHAPE‐Seq v2.0) [42]. Unlike icSHAPE, which includes RNA fragmentation and multiple size selection steps [36], SHAPE‐Seq only requires paired‐end short reads with the site of SHAPE modification and barcode for RNA identity located on opposite ends [41]. While icSHAPE and SHAPE‐seq rely on read termination at the 2′‐O‐adducts sites, SHAPE and mutational profiling (SHAPE‐MaP) utilises the propensity of RT enzymes to read through and incorporate a noncomplementary nucleotide or a deletion at the sites of 2′‐O‐adducts [43, 44, 45]. Reverse transcription can be performed with sequence‐specific or random primers, enabling analysis of either selected RNA species or whole transcriptomes, respectively. SHAPE‐MaP is independent of the sequencing platform but requires high base calling accuracy and coverage. The nucleotide reactivities measured as RT termination or mutation sites are then used as constraints in secondary structure prediction algorithms such as SuperFold [44] to calculate partition functions of probable base pairs, which can be illustrated as arc plots and used to calculate Shannon entropies to identify regions that likely occupy a single, well‐determined conformation.

Most human genes generate transcript isoforms via alternative splicing, transcription start and polyadenylation sites. Short‐read sequencing approaches cannot unambiguously map isoforms due to the fragmentation of the RNA, resulting in sequencing reads that rarely span the entire length of the transcripts. Short‐read SHAPE methods also have trouble resolving long‐range interactions. NanoSHAPE employs nanopore long‐read direct RNA sequencing to probe RNA structure in full‐length transcripts [38]. SHAPE modifications are detected as changes in current signals and extended dwell times as the modified nucleotides pass through the nanopore. NanoSHAPE requires the use of 1‐acetylimidazole (AcIm), which generates compact 2′‐O‐adducts since bulky adducts can cause incomplete translocation, leading to premature read termination. Poor resolution of reactivity at the 5′‐end of RNA due to incomplete reverse transcription, coverage bias inherent to the 3′‐5′ read direction, and the difficulty of translocation through highly structured RNA remain as challenges in NanoSHAPE. Nanopore dimethyl sulphate mutational profiling (Nano‐DMS‐MaP) utilises nanopore direct cDNA sequencing for RNA structure determination by mutational profiling [46]. Similar to other MaP techniques, the modified RNA is reverse transcribed into cDNA with mutations incorporated at modification sites. Nano‐DMS‐MaP uses an ultraprocessive RT to generate cDNA molecules spanning entire transcripts. Structural information is retrieved by mapping mutations introduced by RT upon encountering modified adenosine and cytidine nucleotides caused by the DMS treatment. Despite high intrinsic error rates of nanopore sequencing, the analytical workflow developed for Nano‐DMS‐MaP enables the detection of DMS signals over background noise and structural determination of individual transcript isoforms.

DMS‐seq has also been recently adapted for the detection and characterisation of structures in nascent RNA molecules. Cotranscriptional structure tracking (CoSTseq) measures base pairing as a function of the RNA polymerase position [2]. The cells are permeabilised and then treated with biotinylated CTP in run‐on reactions. The incorporated biotinylated nucleotides mark the 3′‐ends of nascent RNA molecules and the RNA polymerase position. The cells are subsequently treated with DMS, leading to methylation of unpaired nucleotides (single‐stranded RNA regions). Nascent RNA molecules can be purified using streptavidin beads and reverse transcribed using an RT introducing mutations upon encountering methylated sites. High‐throughput sequencing is used to decode both the RNA polymerase position (read ends) and the structure of nascent RNAs (mutation events).

### Methods to study tertiary structures of RNA

RNA structure determination by chemical probing methods such as SHAPE‐ and DMS‐MaP mainly reflects the secondary structures of RNA. However, higher order three‐dimensional structures determined by intra‐ and intermolecular RNA‐RNA, RNA‐protein and protein–protein interactions represent another layer of RNA packaging and function (reviewed in [47]). These interactions are highly dynamic in nature and therefore are more difficult to study in the cellular context. Probing *cis* and *trans* RNA‐RNA interactions with so‐called duplex methods can be utilised to computationally infer RNA tertiary structures [48]. RNA *in situ* conformation sequencing (RIC‐seq) leverages formaldehyde crosslinking to map RNA‐RNA interactions (Fig. 1D) [49]. The crosslinked cells are permeabilised, followed by fragmentation of RNA with nucleases. The 3′‐ends of RNAs are labelled with biotinylated CTP and dephosphorylated while the 5′‐ends are phosphorylated. The resulting biotinylated RNA fragments can be ligated under non‐denaturing conditions, enriched with streptavidin beads and sequenced to obtain chimeric reads representing interactions between RNA fragments located within close proximity. Hence, this method allows global profiling of intra‐ and intermolecular interactions and can be used to generate tertiary structure maps of RNA.

Psoralen analysis of RNA interactions and structures (PARIS) uses a highly specific and reversible crosslinker psoralen‐derivative 4′‐aminomethyltrioxsalen (AMT) to create covalent bonds between RNA base pairs in living cells upon photoactivation [50]. After crosslinking, the RNA is purified, fragmented with RNase, and base‐paired double‐stranded RNA (dsRNA) fragments are isolated with two‐dimensional electrophoresis (native first dimension and denaturing second dimension). The duplex RNA fragments are ligated, and the crosslinking is subsequently reversed to obtain single‐stranded RNA (ssRNA) for sequencing. By mapping the reads, PARIS allows modelling of intra‐ and intermolecular interactions and has been applied to discover higher order structures such as pseudoknots. Other similar techniques using psoralen‐based crosslinkers have also been developed, introducing variations in the crosslinking, RNA fragmentation, proteinase treatment and ligation steps [51, 52, 53, 54].

Kethoxal‐assisted RNA‐RNA interaction sequencing (KARR‐seq) takes advantage of N3‐kethoxal‐mediated RNA labelling [55]. Kethoxal is a cell‐ and nucleus‐permeable small molecule that introduces azide tags to RNA molecules that are substrates for click chemistry. Commercially available dendrimers modified with multiple dibenzocyclooctane (DBCO) and biotin moieties are then used to obtain the crosslinks between RNA molecules. The unique feature of KARR‐seq is the possibility to tune the size of the crosslinkers to probe interactions at different molecular distances. The crosslinked and biotinylated RNA is then fragmented and pulled down with streptavidin beads, followed by repair and ligation to obtain chimeric RNA molecules as described in RIC‐seq and PARIS. RNA‐RNA intra‐ and intermolecular interactions can be deduced from short‐read sequencing, providing a comprehensive RNA contact map.

Tertiary structures of single RNA molecules can also be inferred from chemical probing experiments using a recently developed computational approach based on deconvolution and annotation of ribonucleic conformational ensembles (DANCE‐MaP) [56]. In single‐molecule MaP experiments, each sequencing read represents a structural snapshot of an RNA. However, single RNA molecules can fold into multiple structures, generating different MaP reactivities for the same nucleotides. By using a maximum likelihood (ML) strategy, DANCE‐MaP allows the deconvolution of ensembles and the detection of base pairs and tertiary interactions.

## Functional interplay between RNA structure and RNA‐protein interactions

The development of high‐throughput methods to map transcriptome‐wide cellular RNA structures (2.2–2.3) has facilitated our understanding of the extent of RNA folding in cells, demonstrating that dynamic RNA structures can regulate gene expression by various means (reviewed in [7]). While RNA starts forming base pairing interactions as soon as the nascent RNA emerges at the site of transcription [2], RNA‐binding proteins mediating critical RNA processing steps also engage with RNA cotranscriptionally, forming RNPs [57, 58]. RNA interactome capture studies (2.1) have expanded the RNA‐binding proteome to well over a thousand proteins that have a capacity to bind to RNA [23, 24, 25, 26, 28, 29]. Most notably CLIP, but also other high‐throughput methods (2.1), have enabled the high‐resolution mapping of RBP sites in RNA populations, although they do not reveal the protein composition of individual RNA species. Single‐molecule and other imaging modalities have provided glimpses into the composition of individual RNPs, and these data, together with the high‐throughput studies, have led to an estimate that messenger RNPs are ~40–60% protein by weight [1]. Although RBPs bind specific sequence motifs in the RNA, the presence of a motif alone does not guarantee binding. Access to the motif can be blocked by RNA folding, and the extent to which it is occluded by RNA secondary structure can be a major determinant of RBP binding [59]. While evidence is accumulating that RNA secondary structures could modulate RNA processing by occluding or exposing RBP sites, or by bringing distant sequence elements to close proximity (reviewed in [7, 60, 61, 62, 63, 64]), less is known about the role of RNA‐protein interactions in modifying RNA structures. Here, we discuss some examples of how the methods described in Section 2, together with mechanistic *in vitro* studies, have contributed to our understanding of the complex interplay between RNA structure and RNA‐protein interactions in gene regulation.

### RNA structure and RNA–protein interactions regulate microRNA processing

**Fig. 2:**
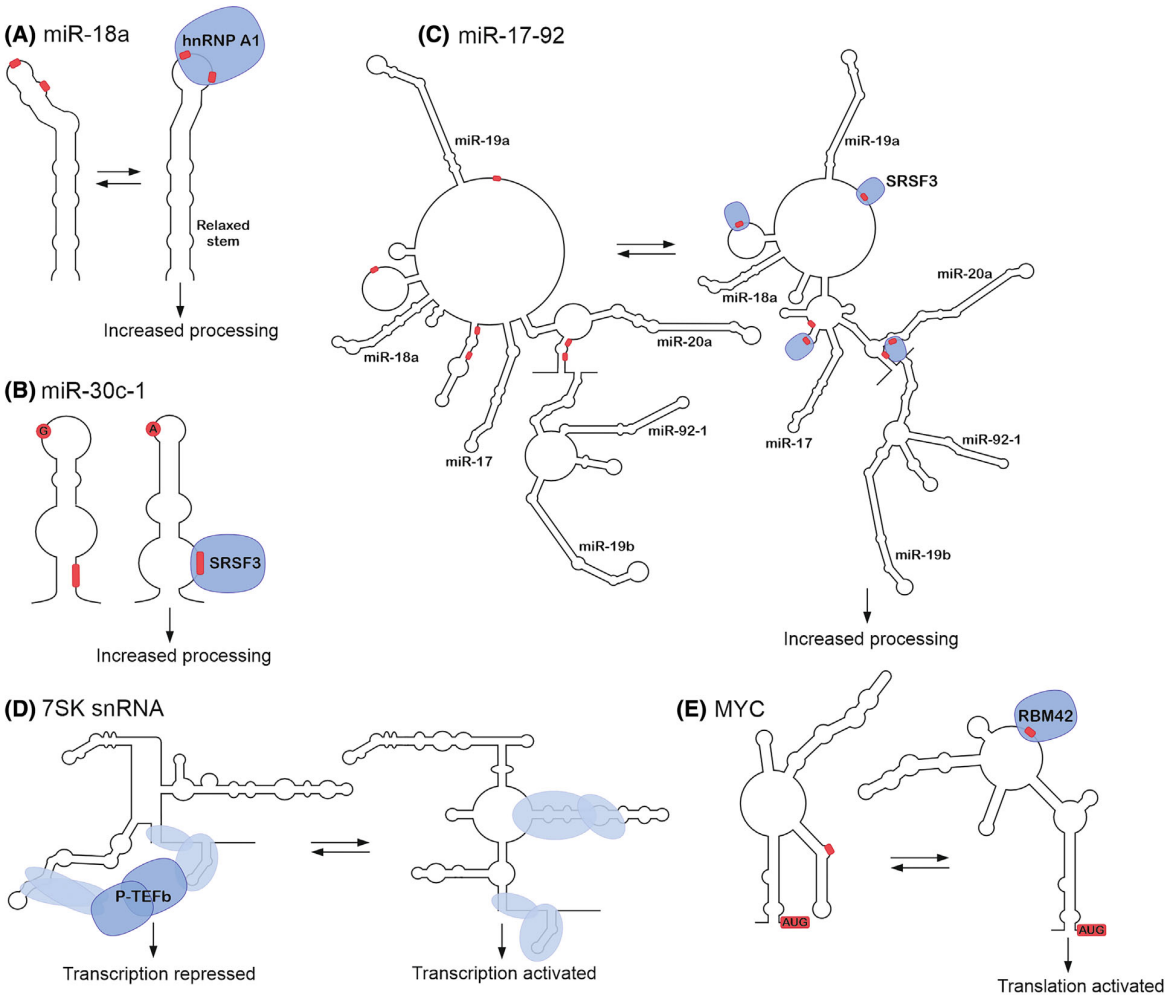
Interplay between RNA structure and RNA‐protein interactions illustrated as predicted RNA secondary structures based on experimental probing data. (A) Heterogeneous ribonucleoprotein A1 (hnRNP A1) binds to paired UAG motifs (in red) within the terminal loop of pri‐miR‐18a, altering the pri‐miRNA conformation and increasing pri‐miRNA processing. (B) A G>A point mutation within the terminal loop of pri‐miR‐30c‐1 induces a secondary structure in which a CNNC motif becomes accessible for Serine/arginine‐rich splicing factor 3 (SRSF3) binding. (C) SRSF3 binds to multiple CNNC sites within the miR‐17‐92 microRNA (miRNA) cluster, inducing a change in the global secondary structure and enhancing the processing of the cluster, particularly miR‐17 and miR‐20a. (D) An allosteric switch triggers a change between conformational states of 7SK, one inducing Positive transcription elongation factor b (P‐TEFb) binding to repress transcription and the other stimulating P‐TEFb release to activate transcription. (E) RNA‐binding motif protein 42 (RBM42) binds to the 5′‐UTR of *MYC* mRNA (only stem III shown) inducing an RNA structure that promotes translation initiation.

MicroRNA precursors form stable hairpin structures that are important for their processing by the Microprocessor complex, composed of the RNaseIII enzyme Drosha and DiGeorge syndrome critical region 8 (DGCR8) [65, 66, 67, 68]. Two major families of splicing factors, SR proteins and heterogeneous ribonucleoproteins (hnRNPs), have been shown to enhance the activity of Microprocessor through mechanisms involving RNA structure modulation [69]. In the case of hnRNP A1, its two RNA recognition motifs (RRMs) mediated the enhanced processing of miR‐18a following hnRNP A1 upregulation [70, 71]. Electrophoretic mobility shift and isothermal titration calorimetry assays revealed that hnRNP A1 specifically interacted with the terminal loop region of pri‐miR‐18a through binding of the RRMs to paired UAG motifs (Fig. 2A). The crystal structure of the hnRNP A1/pri‐miR‐18a complex and nuclear magnetic resonance (NMR) titrations confirmed the interactions. *In vitro* SHAPE experiments with pri‐miR‐18a and increasing amounts of purified hnRNP A1 showed a decrease in reactivity at the terminal loop and, together with site‐directed mutagenesis analysis, suggested that the binding of hnRNP A1 led to an altered conformation of the pri‐miRNA in the proximity of the Drosha cleavage site, thereby increasing the rate of pri‐miRNA processing [71].

A single G>A point mutation within the terminal loop of pri‐miR‐30c‐1 in breast and gastric cancer patients has been linked to increased expression of the mature miR‐30c (Fig. 2B) [72]. *In vitro* SHAPE showed that the mutation induced a secondary structure rearrangement of pri‐miRNA, while hydroxyl radical cleavage footprinting revealed that the terminal loop and nucleotides from +17 to +22 at the 3′‐basal region become solvent accessible in the mutant. The altered structure facilitated the binding of Serine/arginine‐rich splicing factor 3 (SRSF3), a known regulator of pri‐miRNA processing [73, 74], to a CNNC motif (nucleotides from +16 to +19) at the region made accessible. This was in line with *in vitro* studies demonstrating that SRSF3 can interact with CNNC motifs that contain at least one unpaired nucleotide but cannot bind to fully base‐paired CNNC motifs [75]. Single molecule optical tweezer and NMR experiments argue that pri‐miR‐30c‐1 wild‐type and G>A mutant adopt highly similar structures, both of which are bound by SRSF3 [76]. The data suggest that *in vitro*, both variants exist in equilibrium between a monomeric hairpin structure and a kissing hairpin dimer (where the loops of the two hairpins form a dimer), but the dimer of the G>A variant is more unstable, exposing an hnRNP A1 binding site in the loop and facilitating the processing of the monomeric pri‐miR‐30c. This example highlights how a single nucleotide genetic variant can alter the mature levels of miR‐30c‐1 by changing RBP motif accessibility and RNA structure. How these two alternative models translate to the cellular context remains to be demonstrated but highlights the complex interplay between pri‐miRNA structure and interactions with multiple RBPs.

While no methods can directly measure how RNA‐protein interactions affect local and distant base pairing interactions in cells, the comparison of in‐cell SHAPE reactivities to *in vitro* data can be used to assess this [77]. The effect of an individual RBP on RNA secondary structures can also be deduced from differences in‐cell SHAPE reactivities between experimental conditions such as before and after RBP depletion [45, 78]. SRSF3 iCLIP revealed binding to multiple CNNC sites within the miR‐17‐92 miRNA cluster comprising six hairpins, the binding leading to enhanced processing of the cluster miRNAs, particularly miR‐17 and miR‐20a [78]. Comparative SHAPE‐MaP before and after SRSF3 depletion showed differences in local and distant base pairing interactions in an RS‐domain‐dependent manner, suggesting that SRSF3 binding induced changes in the miR‐17‐92 secondary structure and that RS–domain interactions between SRSF3 molecules may play a role in miR‐17‐92 folding and Microprocessor access/cleavage (Fig. 2C). The recent cryogenic electron microscopy (cryoEM) structure of Microprocessor in complex with pri‐let‐7f1 and SRSF3 showed that SRSF3 positioned Drosha onto the basal junction of pri‐let‐7f1 [79]. The intrinsically disordered RS domain of SRSF3 was not visible in the structure; thus, the potential role of the RS domain interactions is an interesting area of future research as SRSF3‐binding sites are found in > 60% of conserved pri‐miRNAs including many clustered miRNAs [74, 80].

### RNA structure and RNA–protein interactions in transcription and translation

Many RNA structures are dynamic and RNAs exist in an equilibrium between several structures. In cells, RNAs may change their conformation in response to protein binding. The RNA component of the 7SK small nuclear ribonucleoprotein (snRNP) functions by sequestering the positive transcription elongation factor b (P‐TEFb) [81, 82]. The binding and release of P‐TEFb involve conformational changes in the structure of 7SK RNA [83, 84]. DANCE‐MaP, the deconvolution and annotation of conformational ensembles, enabled the discovery of two main conformational states of 7SK RNA functioning as an allosteric switch to induce binding of P‐TEFb (Fig. 2D) [56]. The study proposed that release factors, including helicases and other RBPs, trigger a switch between two structural states, thus allosterically stimulating the release of P‐TEFb and inducing transcription. The equilibrium between the structures can be modulated by the proliferative state (transcriptional demand) of the cells, providing an example of RNP structure that functions as an integrator of cellular signals.

Regulation by RBP binding through RNA structures is not limited to noncoding RNAs. A whole genome CRISPRi screen conducted in pancreatic ductal adenocarcinoma (PDAC) cells containing a *MYC*‐5′‐UTR‐GFP fluorescent reporter construct identified an RBP called RNA‐binding motif protein 42 (RBM42) as a selective regulator of *MYC* mRNA translation initiation [85]. *MYC*‐5′‐UTR‐GFP fluorescence and endogenous MYC protein levels were reduced in RBM42 knockout cells without an effect on *MYC* mRNA levels or global translation. Depletion of RBM42 in PDAC cell lines also resulted in decreased cell growth and colony formation that were rescued by MYC expression, indicating that RBM42 was an oncogenic factor acting through MYC. DMS‐seq of the endogenous *MYC*‐5′–UTR revealed a complex secondary structure containing three stems and a large loop at the 3′‐end juxtaposed to the start codon (Fig. 2E). Following RBM42 knockout, the DMS reactivity decreased significantly in the region close to the start codon, indicating a more closed hairpin structure with reduced translation efficiency. CLIP‐seq experiments for RBM42 showed specific binding of RBM42 to the large loop that was remodelled in knockout cells, demonstrating that RBM42 directly bound to the 5′–UTR inducing an RNA structure that promoted active translation.

### RNA structure–protein interactions in drug design

Targeting RNA structures using small molecules represents an emerging therapeutic strategy to modulate gene expression in diseases [86], often by altering RNA‐protein interactions associated with the targeted RNA structure. This approach was recently used to target oncogenic protein synthesis of androgen receptor (AR) and hypoxia‐inducible factor 1A (HIF1A) in prostate cancer [87]. The selective translation eukaryotic initiation factor 4A (eIF4A) inhibitor zotatifin was found to reduce the translation of *AR* and *HIF1A* mRNAs, leading to reduced protein levels with no change in the mRNA abundance. The helicase activity of eIF4A was shown to unwind complex 5'‐UTR secondary structures of *AR* and *HIF1A* mRNAs during translation initiation. DMS‐MaP revealed specific alterations in the 5′‐UTR structure of *AR* and *HIF1A* mRNAs following zotatifin treatment, suggesting that the drug interfered with the 5′‐UTR structure required for translation initiation by eIF4A. The inhibitor was also found to effectively reduce the translation of the constitutively active AR‐v7 androgen receptor splice variant that is associated with castration‐resistant prostate cancer and shares the same 5′‐UTR, suggesting that zotatifin could represent a novel therapeutic agent for the treatment of advanced disease.

The idea of targeting of RNA‐protein interactions with small molecules to regulate gene expression is attractive with extensive clinical potential. While targeting RNA with nucleotide‐based agents has been quite successful including US Food and Drug Administration (FDA)‐approved drugs, the discovery and characterisation of small molecules targeting RBPs has proven to be more challenging (reviewed in [88]). RNA pull‐down confocal nanoscanning (RP‐CONA) method utilising fluorescent on‐bead screening of small molecules binding to RBPs identified the small molecule quercetin that could disrupt the interaction between HuR (human antigen R) and the terminal loop of pri‐miR‐7‐1 [89]. The ability to modulate mature miR‐7 levels by interfering with the HuR‐pri‐miR‐7‐1 interaction could have therapeutic potential as the main target of mature miR‐7 is the mRNA encoding α‐synuclein that plays a role in Parkinson's disease. The same approach was recently used to identify additional HuR inhibitors further demonstrating the feasibility of targeting distinct RBPs and RNA‐protein interactions [90]. Other examples of disease‐associate RBPs with identified small molecule inhibitors include the spliceosome component Splicing factor 3B subunit 1 (SF3B) involved haematological malignancies and the miRNA‐binding proteins LIN28A and B associated with many cancers [88].

The interactions of RNA conformations with RBPs also provide an opportunity to design synthetic RNA molecules that modulate the activity of disease‐relevant protein targets and interfere with their pathological functions. RNA aptamers have emerged as promising candidate RNA drugs due to their high specificity and stability. They are single‐stranded RNA molecules that fold into specific three‐dimensional structures, enabling high‐affinity binding to target proteins. RNA aptamers are commonly discovered using Systematic Evolution of Ligands by Exponential Enrichment (SELEX) [91], where a pool of oligonucleotides with random sequences and structures is incubated with the target proteins, and the best binders are selected based on their affinity in multiple selection rounds. More recently, bioinformatics approaches have been employed to predict the binding propensity of protein–RNA pairs based on secondary structure, hydrogen bonding and Van der Waals interactions, which are then used for *in silico* design of aptamers [92, 93, 94]. RNA aptamers have been used to target oncogenic proteins in cancer (reviewed in [95]) and disease‐causing proteins in neurodegenerative diseases (reviewed in [96]), as both therapeutic and diagnostic tools.

## Conclusions and future perspectives

AlphaFold can predict the three‐dimensional structure of proteins from their amino acid sequence by utilising artificial intelligence (AI) that has greatly accelerated protein‐based discovery research and drug development [97]. The development of AlphaFold was possible because ~100,000 experimental protein structures deposited in structure databases were available to train the deep learning algorithms. Predicting cellular RNA structures faces many challenges. RNA structures are often dynamic, forming an equilibrium between alternating conformations. Furthermore, RNAs form RNPs in the cell where various cellular interactions affect their folding. The past decade has provided tools to map RNA‐protein interactions and RNA structure in the cellular context. The integration of the cellular data with biochemical *in vitro* approaches including high‐resolution cryoEM structures of RNPs has started to accumulate details of the dynamic RNPs in the cell.

Gene regulation via RNPs has a wide impact on human health as exemplified in Section 3. Gene expression may be modulated by therapeutically targeting RNA structure–protein interfaces to stabilise or inhibit the interactions. As exemplified in Section 3.3, such small molecules have already been developed, and they may become clinical reality in the future. RNA secondary and tertiary structures also play important roles in the life cycle of many human pathogens, representing novel targets for drug discovery. Highly complex RNA molecules are often found in RNA viruses where both primary sequence and structures provide information. Comprehensive SHAPE‐MaP profiling of both *in vitro* purified and *in vivo* extracted genomic RNA of hepatitis C virus (HCV) revealed 20 well‐folded regions, and tertiary structure prediction identified three pseudoknots [98]. Synonymous mutations designed to break base pairing in the pseudoknots decreased HCV replication, suggesting they bind factors involved in the viral life cycle and could be exploited in drug design. RNA‐protein interactions can give rise to phase‐separated biomolecular condensates that are formed by liquid–liquid phase separation of the proteins and RNAs that become immiscible with the surrounding liquid environment [99, 100, 101]. Highly structured RNAs can alter the composition of RNP aggregates, either through competing interactions between RNA and protein aggregates or protein sequestration by RNA [102]. While nuclear speckles, stress granules, P‐bodies and paraspeckles are examples of cellular condensates that play important roles in RNA synthesis, processing and degradation [103, 104], pathological RNP condensates are associated with many disease conditions such as neurodegeneration (reviewed in [105]). Thus, misfolded RNPs in condensate‐related diseases may open avenues for new types of therapies.

In conclusion, methodological advances in mapping transcriptome‐wide RNA‐protein interactions and RNA structures have started to uncover the potential of RNP conformations in gene regulation. However, the current methods are not able to directly interrogate *in vivo* RNP structures and are limited to probing one interaction modality at a time. The methods can only probe RNA pools and structural ensembles; thus, they fail to capture the dynamic nature of cellular RNPs and the functional interplay between RNA‐protein interactions and RNA structures. While *in vitro* approaches such as single‐molecule imaging and cryoEM have greatly complemented transcriptome‐wide methods, *in vivo* time‐resolved or single‐molecule methods will be required to fully capture the dynamic RNP conformations in cells. As most current studies have been conducted in cell lines, one future challenge is to adapt these methods to living organisms. Despite these limitations, the enhanced understanding of RNA‐protein interactions and RNA structures has revealed competing RNA‐RNA, RNA‐protein and protein–protein interactions that shape the compaction and function of RNPs throughout their lifetime and may provide novel therapeutic targets in various pathological conditions including infectious diseases, neurological disorders and cancers.

## Conflict of interest

The authors declare no conflict of interest.

## Author contributions

M‐LÄ conceptualised the manuscript and acquired funding support. JR designed and made the figures and tables. M‐LÄ, JR and MS wrote the manuscript.
